# Diagnostic Efficacy and Clinical Relevance of Artificial Intelligence in Detecting Cognitive Decline

**DOI:** 10.7759/cureus.47004

**Published:** 2023-10-13

**Authors:** Ali A Mohamed, Oge Marques

**Affiliations:** 1 Neurological Surgery, Charles E. Schmidt College of Medicine, Florida Atlantic University, Boca Raton, USA; 2 Electrical Engineering and Computer Science, Florida Atlantic University, Boca Raton, USA; 3 Biomedical Sciences, Charles E. Schmidt College of Medicine, Florida Atlantic University, Boca Raton, USA

**Keywords:** machine learning, frontotemporal, lewy bodies, parkinson’s, dementia, diagnosis, mild cognitive impairment

## Abstract

Cognitive impairment is an age-associated disorder of increasing prevalence as the aging population continues to grow. Classified based on the level of cognitive decline, memory, function, and capacity to conduct activities of daily living, cognitive impairment ranges from mild cognitive impairment to dementia. When considering the insidious nature of the etiologies responsible for varying degrees of cognitive impairment, early diagnosis may provide a clinical benefit through the facilitation of early treatment. Typical diagnosis relies heavily on evaluation in a primary care setting. However, there is evidence that other diagnostic tools may aid in an earlier diagnosis of the different underlying pathologies responsible for cognitive impairment. Artificial intelligence represents a new intersecting field with healthcare that may aid in the early detection of neurodegenerative disorders. When assessing the role of AI in detecting cognitive decline, it is important to consider both the diagnostic efficacy of AI algorithms and the clinical relevance and impact of early interventions as a result of early detection. Thus, this review highlights promising investigations and developments in the space of artificial intelligence and healthcare and their potential to impact patient outcomes.

## Introduction and background

With a current report of over 700 million people 65 years of age or older in 2020 and an anticipated doubling of the number of people within this age group in the United States by 2050, it is important to consider the current and anticipated prevalence of certain diseases and disabilities [1]. Cognitive impairment is one specific age-associated disorder to consider.

Cognitive impairment, defined as a decline in cognitive function, can be classified based on the severity of cognitive decline, loss of learning and memory capacity, and dependence on conducting activities of daily living [2,3]. Beyond normal age-related manifestations, cognitive dysfunction ranges from mild cognitive impairment (MCI) to dementia. MCI is identified as a decline in memory and other cognitive abilities [4]. Dementia is identified as a more severe neurocognitive impairment, with a decline in memory, problem-solving, language, and other higher-level cognitive abilities [5]. MCI is clinically described as a transitional phase between normal age-related manifestations and dementia [4]. Progression to dementia from MCI has been estimated to occur in 10% of cases annually [6]. The onset of primary dementia follows a slow progression as the underlying pathology takes its course. The main underlying etiologies, and therefore causes of dementia, include Alzheimer’s disease, frontotemporal dementia, Lewy body disease, and Parkinson’s disease. Vascular dementia, traumatic brain injury, Huntington’s disease, stroke, and prion disease represent other neurodegenerative etiologies of progressive cognitive dysfunction and dementia [7-11].

Because of the insidious nature of neurodegenerative disease, many investigations have focused on early diagnosis and intervention in hopes of improving outcomes for patients [12,13]. Studies have identified the role of lifestyle modification, risk factor mitigation, and early pharmacological intervention in the suppression of disease progression [14,15]. The literature continues to suggest that improved diagnostic capacity, allowing for early intervention, is critical in providing patients with the best outcomes [16,17].

Diagnostic tools for cognitive impairment for many etiologies of dementia include neurocognitive testing, imaging, cerebrospinal fluid assessment, and biomarker tracking [13,18]. However, despite the availability of technical diagnostic methods, typical evaluation of dementia is appropriately done in the primary care setting. Diagnosis is based primarily on the patient’s medical history, with input from close family and friends, followed by a cognitive and neurological examination as indicated. Considering the subtle nature of neurocognitive decline, it is reasonable to conclude that screening tools should be established to ensure early detection and treatment. The 2020 US Preventative Services Task Force came to a similar conclusion, noting that there was evidence for establishing high specificity and sensitivity screening tools for the identification of dementia. However, the overall conclusion was that there remains insufficient evidence to balance the harms and benefits of cognitive impairment screening [19].

Despite uncertainty on asymptomatic screening, with a predilection away from doing so, screening patients experiencing memory difficulties and other suggestive symptoms is generally accepted. Beyond screening based on a patient’s medical history, new novel tools have been explored for evaluation, early detection, and intervention. These include eye tracking devices, peripheral blood-based measures/biomarkers, passive assessments of cognition using smartphone and tablet devices, remote cognitive assessments, and repeatable remote brain assessment using electroencephalography (EEG) [20,21]. Artificial intelligence (AI) is a rapidly growing field that intersects with medicine on many fronts. Previous investigations have specifically explored the role of AI in the detection of neurocognitive and neurodegenerative diseases [22,23]. Other investigations suggest that AI can be leveraged to establish a plan of care for at-risk patients with different forms of neurodecline [24-28]. Ultimately, the role of AI in health care and specifically in diagnosing patients with neurodegenerative disorders remains unknown to many clinicians. Furthermore, it is unclear if earlier detection using AI algorithms sufficiently changes patient outcomes. Thus, we present this review to highlight the capacity of AI in the early detection of neurodegenerative disorders and to discuss the evidence regarding early intervention and its impact on patient outcomes. In this review, we aim to clarify if AI’s capacity for early detection is worth the investment of resources based on how impactful early intervention is on patient outcomes. Many investigations have demonstrated suboptimal outcomes, however, the focus of this review will be to highlight promising investigations and developments in the space of artificial intelligence and healthcare and their potential to impact patient outcomes.

## Review

Mild cognitive impairment (MCI)

MCI represents a predementia phase of disease in patients with symptomatic cognitive and functional impairment. Although primarily focused on Alzheimer’s disease being the underlying pathological basis of the intermediate level of impairment, many other neurodegenerative disorders have been implicated for MCI in more recent investigations [29-31].

Several studies have explored the use of AI machine-learning models for the detection of MCI (Table 1). One study specifically leverages the abnormal patterns of emotion and difficulties with facial muscle control found in patients with MCI [32-35]. Fei et al. utilized a Support Vector Machine model for MCI detection in the early stages [36]. They demonstrated a detection accuracy of 73.3%.

**Table 1:** Overview of papers utilizing artificial intelligence for early detection of neurodegenerative disorders. MCI: mild cognitive impairment; AD: Alzheimer’s disease; PD: Parkinson’s disease; LBD: Lewy body dementia; FTD: frontotemporal dementia; SVM: support vector machines; ANN: artificial neural network; CNN: convolutional neural network; MLP: multilayer perceptron; GBM: gradient boosting machine; RUSRF: random under-sampling random forest; DTC-HPT: decision trees classifier hyperparameter tuning; KNN: k-nearest neighbors classifier; LD: linear discriminant; NB: Naïve Bayes; DL: deep learning; RF: random forest; DT: decision trees; ML: machine learning; NPT: non-linear projection trick; MRI: magnetic resonance imaging; PET: positron emission tomography; CT: computerized tomography; EEG: electroencephalogram; SPECT: single-photon emission computed tomography; WSI: whole-slide images

Reference	Disorder	AI model	Training modality	Accuracy
Fei et al. (2022) [36]	MCI	SVM, MobileNet	Facial expressions	73.30%
Kang et al. (2019) [37]	MCI	ANN	NPT data	96.66%
Boettcher et al. (2020) [38]	MCI, AD	SVM	Clinical data	77.17%
Ghoraani et al. (2021) [39]	MCI, AD	SVM	Clinical data	91%
Goenka and Tiwari (2022) [40]	AD	CNN	MRI	98.30%
Almubark et al. (2020) [41]	AD	MLP	Cognitive data	92.98%
Fulton et al. (2019) [42]	AD	GBM, ResNet-50	MRI	99%
Odusami et al. (2022) [43]	AD	DenseNet201, ResNet18	MRI	98.86%
Pan et al. (2020) [44]	AD	CNN	MRI	84%
Hazarika et al (2022) [45]	AD	DNN, DenseNet, Residual Networks, Inception-V1, V2, V3	MRI	90.22%
Mathotaarachchi et al. (2017) [46]	AD	RUSRF	MRI, PET	84%
Naganandhini et al. (2019) [47]	AD	DTC-HPT	MRI	99%
Pekkala et al. (2017) [48]	AD	SVM	MRI, CT, Clinical data	95%
Bron et al. (2014) [49]	AD	Linear SVM	MRI, PET	89-90%
Herzog et al. (2021) [50]	AD	SVM, KNN, LD, NB	MRI	77-93%
Venugopalan et al. (2021) [51]	AD	SVM, KNN, DL, RF, DT	MRI, Clinical data	68-89%
Battineni et al. (2020) [52]	AD	SVM, RF, GBM, AdaBoosting, LR, NB	MRI	95.96-97.58%
El-Sappagh et al. (2021) [53]	AD	RF	MRI	94.40%
Shimoda et al. (2021) [54]	AD	RF, LR, XGBoost	Clinical data	86.3-89.3%
Sabry et al. (2022) [55]	AD	SVM, KNN, LR, LDA	Clinical data	90.1-91.8%
Miltiadous et al. (2021) [56]	AD	SVM, KNN, RF, DT, ANN, NB	EEG	80-99.1%
Danso et al. (2021) [57]	AD	RF, XGBoost	Clinical data	85-87%
Byeon (2020) [58]	AD, PD	RF	MRI	73.30%
Ni et al. (2021) [59]	LBD, AD	ResNet-50	SPECT	71-90%
Bougea et al. (2022) [60]	LBD, PD	SVM, KNN, binomial logistic regression, NB, Ensemble	Clinical data	82.05-91.2%
Boutet et al. (2021) [61]	PD	ML model	MRI	88%
Signaevsky et al. (2022) [62]	PD	CNN	WSI	99%
Juutinen et al. (2020) [63]	PD	SVM, RF, KNN, LR, LDA, NB, Classification tree, Ensemble, Gaussian Kernel	Clinical data	74.1-84.5%
Hu et al. (2021) [64]	FTD, AD	CNN	MRI	89.86-93.45%
Garcia-Gutierrez et al. (2022) [65]	FTD, AD	SVM, KNN, GBM, DT, NB, RF	PET, Clinical data	82.9-85.4%

Kang et al. utilized the Seoul Neuropsychological Screening Battery, a tool commonly used in Korea for cognitive function assessment in patients with neurological disorders, to develop an algorithm for differentiating and therefore detecting cognitive impairment in patients [37]. Their model demonstrated 79% accuracy in the three-way classification of normal cognition vs. MCI vs. Alzheimer’s disease dementia and 96.66% accuracy in the detection of MCI.

Boettcher et al. and Ghoraani et al. both conducted investigations for the detection of MCI using gait data from the dual-task assessment. With the use of a Support Vector Machine and gradient tree boosting machine learning models, they demonstrated an accuracy of MCI detection ranging from 78% to 81.52% [38,39].

With promising developments in AI-based early detection of MCI, it is reasonable to anticipate better outcomes for patients because of the capacity for early intervention. Despite no specific treatment for MCI, there is evidence suggesting the benefits of early intervention. Memory training and cognitive training have both demonstrated improvements in memory functioning and enhanced brain activity on neuroimaging [66-68]. Previous research has also demonstrated the capacity of traditional Chinese medicines to delay the transition to Alzheimer’s disease from MCI [69]. Considering the evidence of improved outcomes with earlier intervention, advancements in AI for the detection of MCI have the potential to play a major role in the overall care of patients experiencing neurocognitive decline.

Alzheimer’s disease (AD)

AD is a neurodegenerative disorder responsible for the majority of cases of adult-onset dementia [70]. Initially presenting with mild symptoms of short-term memory loss, as AD progresses, patients experience a gradual loss of memory, changes in personality, and other changes in brain function including problem-solving, executive functioning, and judgment [71]. The literature regarding the use of AI for the diagnosis of neurodegenerative decline majorly focuses on AD (Table 1). Almost all previous works utilized AI in conjunction with imaging and/or cognitive datasets.

Goenka and Tiwari utilized a deep convolutional neural network technique with the incorporation of regularization algorithms to achieve three-class categorization of AD, MCI, and normal control whole brain volumetric scans [40]. They demonstrated an accuracy of 98.26% in differentiating between the three classes of neurocognition. Another study utilized convolutional neural networks and deep learning for early diagnosis of AD with accuracy ranging from 93.61% to 97% respective to stage classifications of the disorder within the paper [72]. Similarly, Almubark et al. demonstrated a 92.98% accuracy in diagnosing AD using cognitive data with convolutional neural networks [41]. Fulton et al. and Odusami et al. demonstrated nearly 100% accuracy in detection in their investigations for classifying AD stages with DenseNet and ResNet using the clinical dementia ratio and mini-mental state examination tests [42,43]. Many other studies have proposed novel approaches for early diagnosis of AD using magnetic resonance imaging (MRI) [44-53,58], and electroencephalogram (EEG) [38,39,54-57].

Evident by the number of investigations and the demonstration of high accuracy by the many different proposed algorithms, the use of AI as an aid for the early diagnosis of AD by future clinicians is a reasonable expectation as this area of research continues to grow and prove its efficacy. Despite no definitive treatment of AD, there is evidence that early treatment preserves cognition, behavior, and functional independence in patients. Early treatment with donepezil demonstrated cognitive stabilization and improvement in several control trials [73-76]. Others have demonstrated the efficacy of early treatment with cholinesterase inhibitors [77]. Many other treatment options are currently available for mild and severe cases of AD, but more investigations are required to assess their efficacy early on [78]. In either case, with the continuous introduction of new novel therapeutics for managing AD, early diagnosis is becoming increasingly important for optimizing patient outcomes. With this understanding, it is clear that advances in AI in healthcare, with respect to early diagnosis of AD, have the potential to play a major role in the overall management of patients experiencing this major category of neurocognitive and functional decline.

Frontotemporal dementia (FTD)

FTD is commonly underdiagnosed as symptoms of the disease overlap with different psychiatric manifestations. FTD clinically presents with features of behavior deficit, language deficits, and/or executive function decline [79]. Because of the overlap in clinical presentation, FTD is challenging to diagnose as it must be differentiated from AD and other etiologies of neurocognitive decline. Different algorithms have been explored on this front, but each has presented with different levels of difficulty (Table 1). Some investigations have specifically explored deep learning techniques for differentiating between FTD and AD, but the results remain inconclusive [64]. Deep-learning-assisted diagnostic investigations have demonstrated promising findings but are difficult to apply because they rely on expert-level pre-processing [80]. Genetic algorithms have been successful in differentiating between the two, mimicking etiologies of dementia using machine learning [65]. However, it is clear that further research is required before definitively understanding the potential role of AI in the early diagnosis of FTD.

Despite the necessity of growth in the intersection between AI and healthcare for the management of FTD, it is still crucial to understand if an early diagnosis, beyond what clinicians are currently capable of, could provide value to patients. Unfortunately, there is no indication that an early diagnosis would provide a benefit to patients. There are currently no treatment or management options to decrease the progression of FTD. Current management approaches focus on symptomatic treatment using off-label medications [81]. Although efficacious in some circumstances, there is limited control trial evidence supporting the use of these medications. Furthermore, these medications are not expected to play a role in minimizing the progression of the disease because they do not target the underlying pathophysiology of FTD [82-89]. With an unclear understanding of management and treatment options for patients with FTD, it is not expected that the use of AI for early detection will play a relevant role in patient care. However, if novel treatment approaches demonstrate efficacy at early points of disease presentation, the use of AI for early diagnosis should be explored.

Parkinson’s disease (PD)

PD is a neurodegenerative disorder characterized by bradykinesia and the presence of a resting tremor, rigidity, or similar symptom. PD is a progressive disorder but has a slow onset. Patients typically present with tremors, followed by cardinal features of rigidity and bradykinesia [90,91]. Boutet et al. utilized MRI for the implementation of a machine-learning model for the detection of PD [61]. Signaevsky et al. utilized whole slide images for the implementation of a conventional neural network for the detection of PD [62]. Others have developed algorithms for PD detection with the incorporation of clinical data [55,63] and MRI [58]. Accuracy ranges for these investigations were from 74% to 99% (Table 1). Despite a wide range in accuracy across these investigations, reasonable strides have been made to demonstrate the utility of AI in the early detection of PD. To evaluate the relevance AI will have in patient management, the impact of early detection on outcomes of patients with PD needs to be explored.

Previous literature has established that early treatment of PD is crucial in slowing both the progression and symptomatic manifestations of the disease. Targeted for the management of motor and non-motor symptoms, clinical trials have studied the efficacy of early intervention of PD using rasagiline, ropinirole, pramipexole, and rotigotine [92-95]. However, an important and specific consideration must be made regarding the initiation of early intervention in patients with PD. Many therapeutic options for these patients come with a reasonable risk of additional side effects [96,97]. Outside of pharmacological treatment, physical therapy, speech therapy, and exercise are all early interventions for effective symptom management [98].

Considering the efficacy of early pharmacological and non-pharmacological treatment options for patients with PD, early detection plays a relevant role. Because of this, the role of AI in the early detection of PD is clinically relevant and will likely grow in importance as future investigations demonstrate more streamlined and efficient approaches compared to the current diagnostic standard.

Lewy body dementia (LBD)

LBD is a progressive neurodegenerative disorder that clinically presents with features of both dementia and PD. Typically, patients will present with dementia prior to motor signs and visual hallucinations [99]. Similarly to what has been discussed in the case of FTD, LBD is challenging to diagnose because of its clinical similarities to many different etiologies of dementia. Differentiation of LBD from AD has been achieved using deep learning models leveraging medical experience as a concatenation layer [60]. Other investigations have capitalized on machine learning algorithms that rely on easily attainable, non-invasive predictors such as imaging and patient surveys [59,100]. Accuracy for the discussed investigations ranged from 82.05% to 91.2% (Table 1). Although the literature is modest in this subtype of neurodegenerative decline, findings suggest a reasonable role for AI in the early detection of LBD. However, the clinical relevance of early detection depends on the efficacy of early treatment.

Early intervention for patients with LBD is imperative because it is most responsive early in its disease course [101]. The efficacy of early treatment of LBD has been demonstrated with memantine, rivastigmine, olanzapine, and Yokukansan [102,103]. Of the discussed etiologies of neurocognitive decline, early detection with AI may be most beneficial in the context of LBD. Because of the importance of early intervention and treatment, earlier detection of LBD is critical in optimizing outcomes for patients and improving the quality of care.

## Conclusions

The impact of early treatment for different pathologies responsible for cognitive decline emphasizes the importance of early detection for maximizing patient outcomes. The role of AI in the early detection of cognitive decline is becoming increasingly relevant as novel algorithms continue to demonstrate increased levels of efficacy. However, it is important to recognize that many factors, such as differences in data set size and modalities, reduce the reproducibility of the findings of these studies. It is also important to recognize that reported accuracy may not be the best figure to demonstrate the efficacy of the proposed AI models in detecting neurodegenerative disease. In addition, the lack of clinical application makes it unclear how truly feasible and efficacious these models are. Lastly, the role of early detection, and therefore the role of AI algorithms in that space, may not currently be relevant, regardless of model efficacy, in pathologies with no definitive treatment or evidence of improved outcomes with early intervention. Despite this, future investigations should continue to explore the efficacy of early treatment for all pathologies and the role of AI in the early detection of these pathologies to facilitate earlier intervention.
